# Identification of Human Sulfotransferases Active towards Silymarin Flavonolignans and Taxifolin

**DOI:** 10.3390/metabo10080329

**Published:** 2020-08-12

**Authors:** Jiří Vrba, Barbora Papoušková, Pavel Kosina, Kateřina Lněničková, Kateřina Valentová, Jitka Ulrichová

**Affiliations:** 1 Department of Medical Chemistry and Biochemistry, Faculty of Medicine and Dentistry, Palacký University, Hněvotínská 3, 77515 Olomouc, Czech Republic; pavel.kosina@upol.cz (P.K.); katerina.lnenickova@upol.cz (K.L.); jitka.ulrichova@upol.cz (J.U.); 2 Regional Centre of Advanced Technologies and Materials, Department of Analytical Chemistry, Faculty of Science, Palacký University, 17. Listopadu 12, 77146 Olomouc, Czech Republic; barbora.papouskova@upol.cz; 3 Laboratory of Biotransformation, Institute of Microbiology of the Czech Academy of Sciences, Vídeňská 1083, 14220 Prague, Czech Republic; kata.valentova@email.cz

**Keywords:** silybin, silychristin, silydianin, isosilybin, dihydroquercetin, metabolism, sulfation

## Abstract

Natural phenolic compounds are known to be metabolized by phase II metabolic reactions. In this study, we examined the in vitro sulfation of the main constituents of silymarin, an herbal remedy produced from the fruits of the milk thistle. The study focused on major flavonolignan constituents, including silybin A, silybin B, isosilybin A, isosilybin B, silychristin, and silydianin, as well as the flavonoid taxifolin. Using ultra-high-performance liquid chromatography coupled with tandem mass spectrometry (UHPLC-MS), individual flavonolignans and taxifolin were found to be sulfated by human liver and human intestinal cytosols. Moreover, experiments with recombinant enzymes revealed that human sulfotransferases (SULTs) 1A1*1, 1A1*2, 1A2, 1A3, 1B1, 1C4, and 1E1 catalyzed the sulfation of all of the tested compounds, with the exception of silydianin, which was not sulfated by SULT1B1 and SULT1C4. The sulfation products detected were monosulfates, of which some of the major ones were identified as silybin A 20-*O*-sulfate, silybin B 20-*O*-sulfate, and isosilybin A 20-*O*-sulfate. Further, we also observed the sulfation of the tested compounds when they were tested in the silymarin mixture. Sulfates of flavonolignans and of taxifolin were produced by incubating silymarin with all of the above SULT enzymes, with human liver and intestinal cytosols, and also with human hepatocytes, even though the spectrum and amount of the sulfates varied among the metabolic models. Considering our results and the expression patterns of human sulfotransferases in metabolic tissues, we conclude that flavonolignans and taxifolin can potentially undergo both intestinal and hepatic sulfation, and that SULTs 1A1, 1A3, 1B1, and 1E1 could be involved in the biotransformation of the constituents of silymarin.

## 1. Introduction

**Figure 1:**
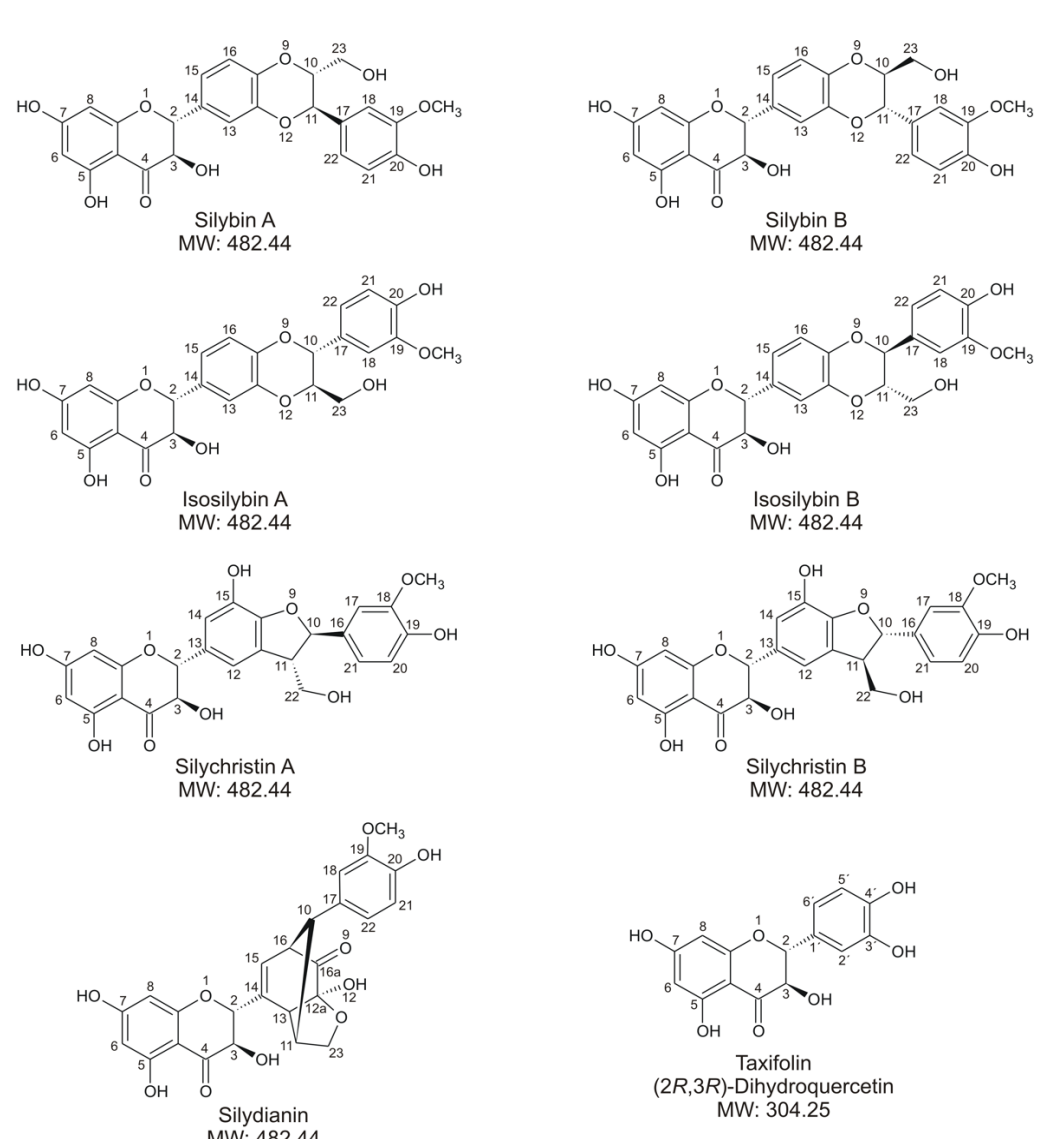
Chemical structures of tested silymarin constituents.

Silymarin is an herbal hepatoprotective remedy produced from the fruits of the milk thistle (*Silybum marianum*). It is a complex mixture consisting primarily of (i) flavonolignans, including mainly silybin A, silybin B, isosilybin A, isosilybin B, silychristin A, silychristin B, and silydianin; (ii) their flavonoid precursor taxifolin, also called (2*R*,3*R*)-dihydroquercetin (Figure 1); and (iii) a fraction of undefined products formed by the oxidation and polymerization of flavonolignans [1,2]. Over the last few decades, silymarin and its constituents have been the subject of a wide variety of biological studies [3,4,5]. Research work has naturally also focused on their metabolism. In the human organism, silymarin constituents are supposed to undergo bioconversion by intestinal microbiota [6,7], or they can be absorbed and metabolized in tissues such as the intestine and liver [8]. The biotransformation processes in human tissues include two types of reactions, classified as phase I (functionalization) and phase II (conjugation) reactions. The most important enzymes of the phase I biotransformation are cytochromes P450 (CYPs) [9]. It has been demonstrated that individual silymarin flavonolignans may be demethylated or hydroxylated by one or more human recombinant CYP enzymes, namely CYPs 2C8, 2C19, 2D6, and 3A4, of which the latter exhibited the broadest substrate specificity towards this class of compounds [10]. The parent flavonolignans are, however, sufficiently functionalized for phase II conjugations without the need for prior phase I reactions. In isolated human hepatocytes, flavonolignans were found to be conjugated mainly by glucuronidation or sulfation, while other types of conjugations, such as methylation, glutathionylation, and cysteine conjugation, as well as the formation of phase I products, were only marginal [10,11]. The conjugations with glucuronic acid or sulfate are also the only metabolic pathways reported so far for silymarin flavonolignans in humans [12,13]. The flavonoid taxifolin, bearing five hydroxyl groups, can also be metabolized directly by phase II conjugations [14,15]. Taxifolin was shown to be converted to a sulfate, methyl derivative, or methylated glucuronide in human hepatocytes [14], and the same types of conjugations were found in rats [16].

Existing research suggests that after the administration of silymarin, human tissues are exposed to the conjugated forms of its constituents. Therefore, there has been increasing scientific attention on identifying the enzymes involved in the conjugation processes, the preparation of the conjugates, and their biological evaluation [8]. It may be presumed that UDP-glucuronosyltransferases (UGTs), sulfotransferases (SULTs), and catechol *O*-methyltransferases play the main roles in the biotransformation of silymarin flavonolignans and taxifolin. To date, the activity of human UGT enzymes has only been investigated on the main silymarin flavonolignans [11,17,18], with UGTs 1A1, 1A3, 1A8, and 1A9 having been found to be particularly important for flavonolignan glucuronidation [11]. In this study, we examined the in vitro sulfation of silymarin flavonolignans and taxifolin and aimed to identify human sulfotransferases that are active towards the tested compounds.

## 2. Results

### 2.1. Sulfation of Flavonolignans and Taxifolin by Human Tissue Cytosols

The tested silymarin constituents and their biotransformation products were analyzed by the UHPLC-MS method using a C18 column and gradient elution. The parent flavonolignans (*m/z* 481.115) were eluted at retention times ranging between 6.0 and 7.6 min (Table 1), in the order of silychristin A, silydianin, silychristin B, silybin A, silybin B, isosilybin A, and isosilybin B. Silychristin A and silychristin B, present in the tested sample of silychristin in the ratio of 7.6:1 (A/B) according to the peak areas, were identified in the chromatograms on the basis of previously published retention times [19]. The flavonoid taxifolin (*m/z* 303.050) was eluted before the flavonolignans. The tested sample of taxifolin gave two peaks with *m/z* 303.050 at 4.4 and 4.9 min (Table 1), respectively, with a peak area ratio of 22:1. The main peak was attributed to taxifolin, while the minor one was assumed to be a dihydroquercetin isomer with a configuration other than (2*R*,3*R*).

Using the UHPLC-MS method, the study first investigated the sulfation of individual constituents of silymarin by human liver and intestinal cytosols. The analyses demonstrated that the incubation of both cytosolic fractions for 30 min with individual flavonolignans or taxifolin (50 µM) resulted in the formation of at least one sulfated metabolite with each of the tested compounds (Table 1). All of the metabolites produced were monosulfates, while the chromatographic peaks corresponding to flavonolignan monosulfates (*m/z* 561.069) and taxifolin monosulfates (*m/z* 383.006) were consistently found at lower retention times than the respective parent compounds. The experiments also showed that the intestinal cytosol had a higher sulfation activity than the liver cytosol. The fractions of sulfated metabolites produced by human liver cytosol only reached 0.1–0.8%. With human intestinal cytosol, the sulfation was between 0.4 and 4.9% for flavonolignans, while it reached 14.3% for taxifolin. According to the percentage conversion, the susceptibility of the tested compounds to sulfation by the intestinal cytosol was in the following order: taxifolin > isosilybin A ≈ silychristin A/B ≈ isosilybin B > silydianin ≈ silybin B ≈ silybin A (Table 1).

### 2.2. Sulfation of Flavonolignans and Taxifolin by Human Sulfotransferases

The study further focused on the identification of human sulfotransferases that exhibit sulfation activity towards the tested silymarin constituents. For that purpose, flavonolignans and taxifolin (50 µM) were individually incubated for 30 min with nine recombinant, commercially available human SULT enzymes. Using the UHPLC-MS analyses, we found that each of the tested compounds was sulfated by multiple human SULTs (Table 1). The sulfation resulted solely in the formation of monosulfated products, which mostly corresponded to those obtained with the tissue cytosols. Under the given conditions, most of the tested SULT enzymes produced a higher number or higher amounts of the sulfated metabolites than the tissue cytosolic fractions. We found that silybin A, silybin B, isosilybin A, and isosilybin B were each converted into two relevant sulfation products, and with the exception of isosilybin A, to another less relevant sulfation product. Silychristin A/B was converted into one major and three minor sulfates, while a single sulfation product was found with silydianin. Using available standards of sulfated flavonolignans, we identified silybin A 20-*O*-sulfate, silybin B 20-*O*-sulfate, and isosilybin A 20-*O*-sulfate to be produced as the main sulfates of the respective parent flavonolignans by certain SULT enzymes, and by human liver and intestinal cytosols (Table 1). The incubations with taxifolin gave the highest number, namely six potential sulfated metabolites, but at least one of them (t_R_ 4.5 min) was assumed to be a product of a dihydroquercetin isomer, since it was eluted later than taxifolin. The six taxifolin sulfates included three major and three minor ones, with one of the minor products being identified as taxifolin 4′-*O*-sulfate. Consistent with the results obtained with the intestinal cytosol, taxifolin was more susceptible to the SULT-catalyzed sulfation than flavonolignans. The maximum fractions of the sulfated metabolites found for individual compounds with the respective SULT enzymes exhibited the following hierarchy: taxifolin (> 99%) > silydianin (92%) ≈ silychristin A/B (90%) > silybin B (86%) ≈ silybin A (86%) > isosilybin A (81%) > isosilybin B (66%) (Table 1).

As summarized in Table 2, SULTs 1A1*1, 1A1*2, 1A2, 1A3, and 1E1 were found to catalyze the sulfation of all of the tested compounds. SULT1B1 and SULT1C4 also sulfated all the tested compounds, with the exception of silydianin, which only gave a very low conversion. On the other hand, SULT1C2 and SULT2A1 exhibited no or almost negligible activity towards all of the tested silymarin constituents. Among the active enzymes, SULT1E1 produced the highest amounts of the sulfated metabolites of both silybin A and silybin B. The second most active enzymes for silybin A and silybin B were SULT1C4 and SULT1A3, respectively, but these enzymes preferentially produced different sulfates than SULT1E1. The most active enzymes for the sulfation of isosilybin A were SULT1C4 and SULT1A3, while the sulfation of isosilybin B was primarily catalyzed by SULT1A3 and SULT1E1. Further, silychristin A/B was mainly sulfated by SULTs 1A3, 1C4, and 1E1; silydianin was mainly sulfated by SULTs 1E1, 1A1*1, and 1A1*2; and the highest amounts of taxifolin sulfates were produced by SULT1A3 and SULT1C4 (Table 2).

It is worth noting that we did not find any substantial differences between the sulfation catalyzed by SULT1A1*1 and SULT1A1*2, which are the most common allozymes of SULT1A1 [20]. On the other hand, there were certain differences in the sulfation of silybin and isosilybin diastereomers A and B. For instance, SULTs 1A1*1, 1A1*2, 1A2, and 1C4 exhibited a higher activity with silybin A, while SULT1A3 was more active with silybin B. Similarly, SULT1B1 and SULT1C4 exhibited a higher activity with isosilybin A, while SULT1A2 and SULT1E1 were more active with isosilybin B (Table 1 and Table 2).

### 2.3. Sulfation of Silymarin Mixture by Human Sulfotransferases

**Figure 2:**
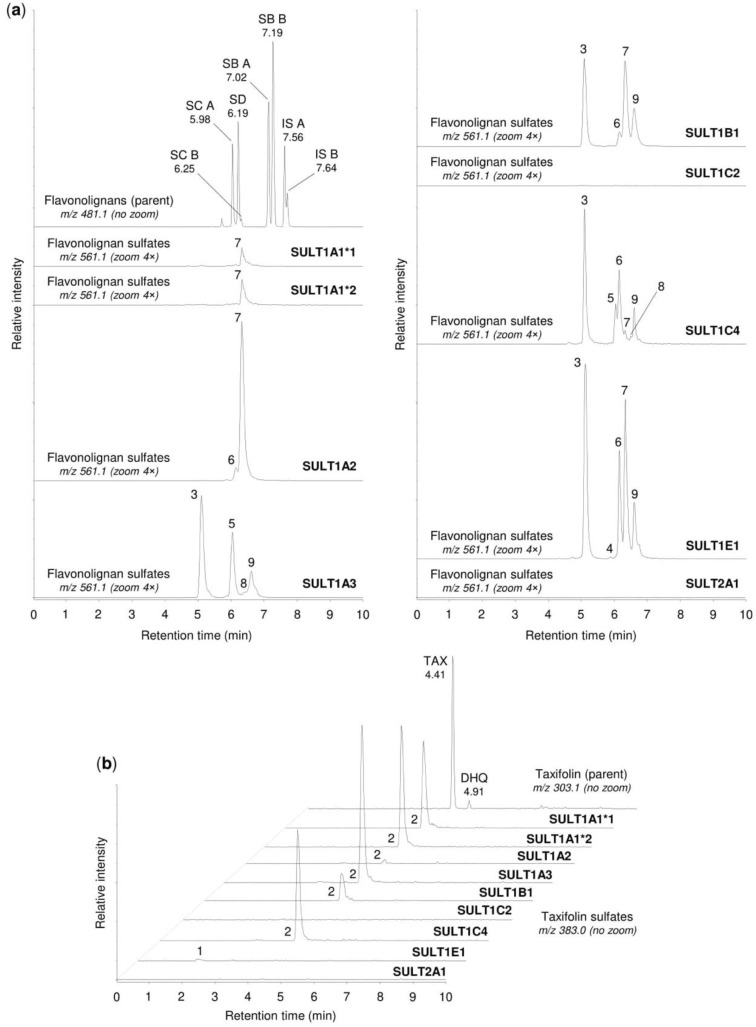
Sulfation of silymarin mixture by human sulfotransferases. Silymarin (50 µM) was incubated for 30 min with recombinant human SULTs 1A1*1, 1A1*2, 1A2, 1A3, 1B1, 1C2, 1C4, 1E1, and 2A1, then analyzed by the UHPLC-MS method. (**a**) The representative chromatograms show the parent flavonolignans, i.e., silydianin (SD), silychristin A (SC A), silychristin B (SC B), silybin A (SB A), silybin B (SB B), isosilybin A (IS A), and isosilybin B (IS B), along with their sulfation products, marked as peaks 3–9. (**b**) The representative chromatograms show the parent taxifolin (TAX), a dihydroquercetin isomer (DHQ), and their sulfation products, marked as peaks 1 and 2. For details of the peaks, see Table 3. To enable comparisons between analyses, the signal intensity in chromatograms was adjusted to the same value within each panel (4.00 × 10^5^ in (**a**), 1.80 × 10^4^ in (**b**)), before using the zoom tool where appropriate.

The study also investigated the sulfation of flavonolignans and taxifolin when tested as their natural mixture, i.e., silymarin. After the incubation of SULT enzymes for 30 min with 50 µM silymarin, we detected a total of nine chromatographic peaks, which also corresponded to the sulfates produced with the individually tested silymarin constituents. Of these peaks, seven were formed by flavonolignan monosulfates (*m/z* 561.069) and two by taxifolin monosulfates (*m/z* 383.006) (Figure 2). The list of the peaks together with potential metabolites, deduced on the basis of the t_R_ values and MS data, is given in Table 3. Of the tested enzymes, SULTs 1A1*1, 1A1*2, 1A2, 1A3, 1B1, 1C4, and 1E1 showed relevant sulfation activity with silymarin, whereas the activities of SULT1C2 and SULT2A1 were negligible. As shown in Figure 2 and Table 2, SULT1A1*1 and SULT1A1*2 allozymes exhibited a comparable catalytic effect with silymarin, while the other active enzymes differed both in the spectrum and amount of sulfates produced. SULT1E1 was found to produce the highest total amount of the sulfated metabolites, which were detected as five peaks at *m/z* 561.069 and one peak at *m/z* 383.006 (Figure 2). On the basis of the sums of the peak areas of the sulfates produced, the following rank order was obtained for the overall sulfation activity with silymarin: SULT1E1 > SULT1B1 ≈ SULT1A3 ≈ SULT1C4 > SULT1A2 > SULT1A1*2 ≈ SULT1A1*1 (Table 2). This rank order fully corresponded to the overall sulfation of the flavonolignan constituents. In contrast, the rank order reflecting the sulfation of taxifolin in the silymarin mixture was different, as follows (the relative activities are given in brackets): SULT1A3 (100%) > SULT1A1*2 (73%) ≈ SULT1C4 (72%) > SULT1A1*1 (59%) > SULT1B1 (20%) > SULT1A2 (2%) ≈ SULT1E1 (2%).

### 2.4. Sulfation of Silymarin Mixture by Human Tissue Cytosols and Human Hepatocytes

**Figure 3:**
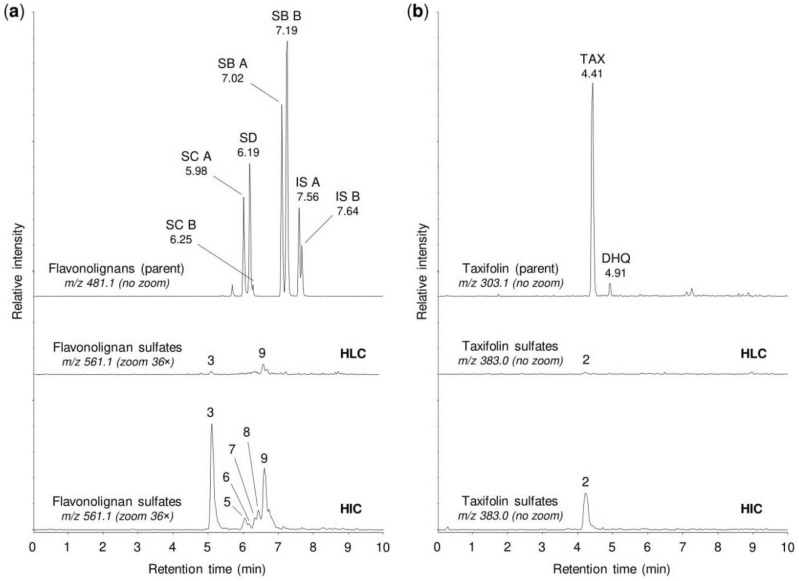
Sulfation of silymarin mixture by human tissue cytosols. Silymarin (50 µM) was incubated for 30 min with pooled human liver cytosol (HLC) or pooled human intestinal cytosol (HIC), then analyzed by the UHPLC-MS method. (**a**) The representative chromatograms show the parent flavonolignans, i.e., silydianin (SD), silychristin A (SC A), silychristin B (SC B), silybin A (SB A), silybin B (SB B), isosilybin A (IS A), and isosilybin B (IS B), along with their sulfation products. (**b**) The representative chromatograms show the parent taxifolin (TAX), a dihydroquercetin isomer (DHQ), and their sulfation products. The peaks of sulfates are marked with numbers corresponding to those in Figure 2. For details on the peaks, see Table 3. To enable comparisons between analyses, the signal intensity in chromatograms was adjusted to the same value within each panel (3.05 × 10^5^ in (**a**), 1.00 × 10^4^ in (**b**)), before using the zoom tool where appropriate.

As expected, the sulfation of the tested silymarin constituents also occurred by incubating 50 µM silymarin for 30 min with human tissue cytosols. After the incubation with human liver cytosol, the UHPLC-MS analyses detected only two small peaks at *m/z* 561.069 and another one at *m/z* 383.006, corresponding to the monosulfated forms of flavonolignans and of taxifolin, respectively (Figure 3, Table 3). In contrast, human intestinal cytosol produced a wider spectrum and higher amounts of the sulfated metabolites. The incubation of silymarin with the intestinal cytosol resulted in the appearance of six peaks corresponding to flavonolignan monosulfates (*m/z* 561.069) and one peak corresponding to taxifolin monosulfate (*m/z* 383.006). Of these peaks, the largest one was formed by the sulfates of silychristin A/B or silydianin (peak # 3). Moreover, the sulfation products of isosilybins A and B (peaks # 7–9) predominated over those of silybins A and B (Figure 3, Table 3).

**Figure 4:**
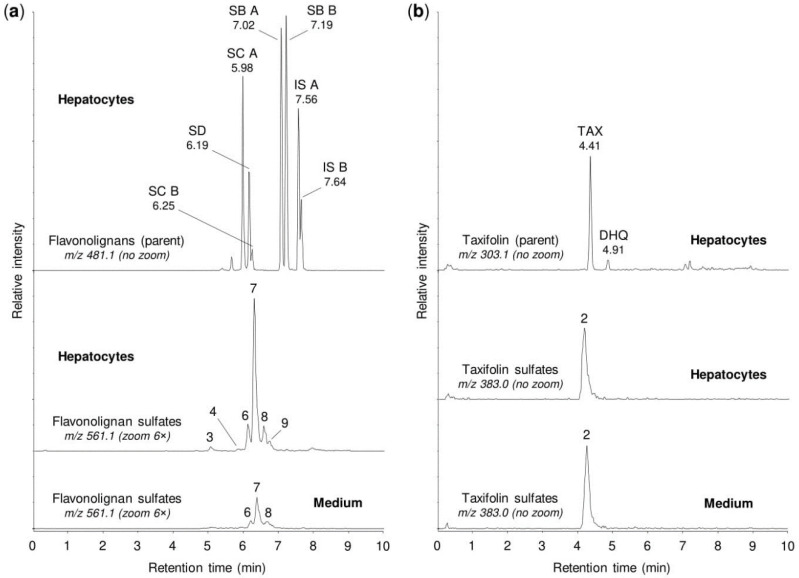
Sulfation of silymarin mixture by human hepatocytes. The suspension of human hepatocytes was incubated for 1 h with silymarin (50 µM), and then the cells and medium were analyzed by the UHPLC-MS method. (**a**) The representative chromatograms show the parent flavonolignans, i.e., silydianin (SD), silychristin A (SC A), silychristin B (SC B), silybin A (SB A), silybin B (SB B), isosilybin A (IS A), isosilybin B (IS B), and their sulfation products. (**b**) The representative chromatograms show the parent taxifolin (TAX), a dihydroquercetin isomer (DHQ), and their sulfation products. The peaks of sulfates are marked with numbers corresponding to those in Figure 2. For details on the peaks, see Table 3. To enable comparisons between analyses, the signal intensity in chromatograms was adjusted to the same value within each panel (3.05 × 10^5^ in (**a**), 1.00 × 10^4^ in (**b**)), before using the zoom tool where appropriate.

Despite the low sulfation activity of the liver cytosol, we found relatively efficient sulfation of the tested silymarin constituents in primary cultures of human hepatocytes (Figure 4, Table 3). After the incubation of silymarin (50 µM) for 1 h with hepatocyte suspensions, the UHPLC-MS analyses of the cells and culture medium revealed up to six peaks corresponding to flavonolignan monosulfates (*m/z* 561.069) and one peak corresponding to taxifolin monosulfate (*m/z* 383.006). We found that all of the tested silymarin constituents were or could be potentially sulfated in human hepatocytes, and the sulfates of isosilybins A and B (peaks # 7–9) were much more abundant than the sulfation products of other constituents (Figure 4, Table 3). In addition, our analyses also demonstrated that human hepatocytes were able to produce other types of flavonolignan metabolites, including monoglucuronides (*m/z* 657.145), hydrogenated derivatives (*m/z* 483.125), and hydrogenated monoglucuronides (*m/z* 659.161).

## 3. Discussion

This study was designed to examine in vitro the metabolic sulfation of the main constituents of silymarin, with the focus on the flavonoid taxifolin and major flavonolignans derived from taxifolin, including silybin A, silybin B, isosilybin A, isosilybin B, silychristin A/B, and silydianin [2]. The study also included silymarin itself, which is used for the treatment of liver disorders, and thus the results could be more relevant for clinical situations. The tested compounds have five or six hydroxyl groups in their structures, and hence they can be readily metabolized by conjugation reactions, such as glucuronidation and sulfation. In xenobiotic metabolism, this type of modification is mostly associated with the inactivation of the target molecules and their elimination from the body [21]. For taxifolin, sulfation was recognized as the main biotransformation pathway in human hepatocytes [14]. Flavonolignans appear to be preferentially glucuronidated, nonetheless their sulfation was, with the exception of silydianin, also reported both in vitro [10] and in humans [13].

In contrast to the parent compounds, sulfated forms of silymarin constituents cannot be isolated from plant material. The only exception is taxifolin 7-*O*-sulfate, which was recently identified in a hybrid species of willow [22]. Several sulfated derivatives were, however, prepared by chemical [23] or enzymatic synthesis using the microbial aryl sulfotransferase from *Desulfitobacterium hafniense* (also see Section 4.1) [24,25,26], the aryl sulfotransferase IV from rat liver [27,28], or the fungus *Cunninghamella blakesleeana* [29]. In this study, we examined the sulfation of silymarin constituents by human enzymes with sulfotransferase activity. There are two classes of human sulfotransferases: (i) membrane-bound enzymes located in the Golgi apparatus, participating in the modification of proteins, lipids, and glycosaminoglycans; and (ii) cytosolic enzymes involved in the conjugation of xenobiotics and small endogenous substrates [20]. The cytosolic sulfotransferases include 13 enzymes that can be found in many tissues [30]. Using commercially available enzymes, we found seven human cytosolic sulfotransferases, SULTs 1A1*1, 1A1*2, 1A2, 1A3, 1B1, 1C4, and 1E1, which catalyze the sulfation of taxifolin and of all of the tested flavonolignans, with one exception—silydianin was not sulfated by SULT1B1 and SULT1C4. In addition, the seven SULT enzymes were also able to produce sulfated metabolites with the silymarin mixture. Of the active enzymes, SULT1A2 is not expressed at the protein level in human tissues, and SULT1C4 is mainly found during fetal development [30]. On the other hand, SULTs 1A1, 1B1, and 1E1 are among the main hepatic sulfotransferases, and together with SULT1A3 they are also expressed in the small intestine [31]. We may, therefore, speculate that SULTs 1A1, 1A3, 1B1, and 1E1 could play a role in the metabolic sulfation of silymarin constituents in humans, and that the sulfation could occur in both the small intestine and in the liver. The latter premise is supported by our observations using human hepatocytes and intestinal cytosol. Moreover, comparison of the sulfation activity of the liver and intestinal cytosols suggests that the intestine could be more important for the sulfation of silymarin constituents than the liver.

It is also worth noting that monosulfated derivatives of the tested compounds were solely produced regardless of the metabolic model used. This suggests that the sulfation of one hydroxyl group in the molecules of flavonolignans and taxifolin may prevent potential sulfation of remaining hydroxyl groups. Finally, as mentioned above, the physiological function of cytosolic sulfotransferases is not restricted to xenobiotic metabolism. For instance, SULTs 1E1, 1A3, and 1B1 are known to catalyze the sulfation of estrogens, catecholamines, and thyroid hormones, respectively [20,30]. Hence, when interpreting the pharmacological effects of silymarin or its individual constituents, it should be taken into account that their interaction with the SULT enzymes could inhibit the biotransformation of some endogenous molecules, and thus potentially interfere with related signaling pathways.

## 4. Materials and Methods

### 4.1. Tested Compounds

For metabolic experiments, silymarin (product no. 9065110) was kindly provided by Indena (Italy). According to high-performance liquid chromatography (HPLC) analysis, this product contained 9.3% silybin A, 13.8% silybin B, 4.9% isosilybin A, 2.1% isosilybin B, 11.0% silychristin A, 2.4% silychristin B, 13.1% silydianin, and 2.8% taxifolin [19]. Individual flavonolignans, i.e., silybin A (100% purity), silybin B (100% purity), isosilybin A (96% purity), isosilybin B (98% purity), silychristin A/B (98% purity), and silydianin (100% purity) were prepared from silymarin, purchased from Liaoning Senrong Pharmaceutical (China), as described previously. For details on their preparation, see the references in [11]. Taxifolin (96% purity) was obtained from Amagro (Czech Republic). For all experiments, 50 mM stock solutions of the individual compounds and silymarin (assuming a molecular weight of 482.44) were prepared freshly before use by dissolving them in dimethyl sulfoxide (DMSO; Sigma-Aldrich, St. Louis, MO, USA).

The standards of possible sulfated metabolites were prepared by sulfation of the respective compounds using the aryl sulfotransferase from *Desulfitobacterium hafniense*. For the preparation of silybin A 20-*O*-sulfate (99% purity), silybin B 20-*O*-sulfate (98% purity), and isosilybin A 20-*O*-sulfate (100% purity), see [24,26]; for the preparation of taxifolin 4′-*O*-sulfate (96% purity), see [25,28]. The purity of individual silymarin constituents and of the sulfated standards was determined by HPLC.

### 4.2. Biotransformation in Human Hepatocytes

The Ethics Committee of University Hospital Olomouc approved the protocols for the preparation and use of human hepatocytes (approval no. 119/07). The hepatocytes used in the study originated from liver samples of three multiorgan donors: a 60-year-old man (culture LH79), a 45-year-old man (culture LH81), and a 56-year-old man (culture LH83). Hepatocytes were isolated using a collagenase perfusion technique, resuspended in serum-free culture medium consisting of Williams’s medium E, Ham’s F-12 medium, and additives, as described previously [32], and immediately used for the experiments. The cell suspensions (4 × 10^6^ cells/mL) were incubated using an ES-20 Environmental Shaker (Biosan, Latvia) for 1 h at 37 °C and 140 rpm with 50 µM silymarin or 0.1% (*v/v*) DMSO (control). After the incubation, the cells and medium were separated by centrifugation for 5 min at 150 × *g* and 4 °C, then frozen at −80 °C. The samples were subsequently analyzed by ultra-high-performance liquid chromatography coupled with tandem mass spectrometry (UHPLC-MS).

### 4.3. Sulfation by Human Tissue Cytosols and Human Sulfotransferases

Sulfation of the tested compounds was examined using human liver and intestinal cytosols, as well as by using individual human sulfotransferases. Human liver cytosol pooled from 50 donors of mixed gender (H0610.C) and human intestinal cytosol pooled from 13 donors of mixed gender (H0610.IC) were obtained from Sekisui XenoTech (Kansas City, KS, USA). Cytosolic fractions from *Escherichia coli* expressing recombinant human SULT1A1*1, SULT1A1*2, SULT1A2, SULT1A3, SULT1B1, SULT1C2, SULT1C4, SULT1E1, and SULT2A1 were obtained from Cypex (Dundee, UK). The incubations were performed in 0.2 mL of potassium phosphate–HCl buffer (pH 7.4; 50 mM) containing 5 mM MgCl_2_, 10 mM dithiothreitol (Sigma-Aldrich, St. Louis, MO, USA), 50 µM tested compounds or silymarin in 0.1% (*v/v*) DMSO, 100 µg/mL tissue cytosol protein or 50 µg/mL *E. coli* cytosol protein, and 120 µM 3’-phosphoadenosine 5’-phosphosulfate (PAPS; Sigma-Aldrich, St. Louis, MO, USA). Control samples were prepared by incubating the tested compounds in the absence of PAPS or the cytosolic fractions. After incubation for 30 min at 37 °C and 300 rpm using a Thermomixer Comfort (Eppendorf, Germany), the samples were stored at –80 °C until their analysis by UHPLC-MS.

### 4.4. UHPLC-MS Analysis of Biotransformation Products

Human hepatocytes collected by centrifugation were resuspended in 0.4 mL of methanol-acetic acid (95:5, *v/v*) and disintegrated by sonication on ice (10 cycles, 0.5 s pulses, 50% amplitude) using a UP200s Ultrasonic Processor equipped with a Sonotrode S2 sonicator probe (Hielscher, Germany). The samples of media and incubation mixtures with the cytosolic fractions were thawed and mixed 1:1 (*v/v*) with methanol-acetic acid (95:5, *v/v*). All samples were centrifuged for 5 min at 12,000 × *g* and 4 °C, and the supernatants were analyzed by UHPLC-MS. The analyses were performed using an Acquity UPLC I-Class system (Waters, Milford, MA, USA), including a solvent manager with a degasser system, sample manager, and column manager with a Kinetex 2.6 µm Polar C18 column (100 × 2.1 mm i.d.; Phenomenex, Torrance, CA, USA). The binary mobile phase consisted of 5 mM ammonium acetate aqueous solution, pH 3 (solvent A), and methanol (solvent B). The flow rate was 0.5 mL/min, and the linear gradient elution program included: 0–2 min 10% B, 2–8 min 10–55% B, 8–8.5 min 55–70% B, 8.5–9 min 70–10% B, followed by equilibration at 10% B. The column oven was maintained at 35 °C, the samples were cooled in the autosampler at 10 °C, and the injection volume was 2 µL.

The electrospray ionization source of a Synapt G2-S Mass Spectrometer (Waters, Milford, MA, USA) operated in negative mode with the following instrument settings: capillary voltage 2.25 kV, sampling cone 35 V, source temperature 120 °C, desolvation temperature 300 °C, cone gas flow 25 L/h, and desolvation gas flow 600 L/h. The data acquisition range was 50–1200 Da with a scan time of 0.2 s and the instrument was calibrated using a sodium formate solution in acetonitrile. Data were automatically processed and corrected for mass error during acquisition with a leucine enkephalin as an external reference. Data were obtained using two interleaved scan functions (MS^E^ experiments), enabling the simultaneous acquisition of both low-collision-energy (4 V) and high-collision-energy (15–30 V) mass spectra from a single experiment. Post-acquisition processing of the data was performed using the software Metabolynx (Waters, Milford, MA, USA). MS data with ppm ≥ 5 were not considered.

The UHPLC-MS method was validated with the parent compounds and available sulfated standards. The method validation included tests of selectivity, linearity, precision, and accuracy. The selectivity was evaluated by comparing the control samples with samples spiked with the tested compounds. The linearity was tested over the concentration range of 0.5–25 µM, and the values of the coefficient of determination (R^2^) were found to be in the range of 0.9969–1.0000. The inter-day precision and accuracy tested in three replicates were expressed as the relative standard deviation (RSD) and relative error (RE), respectively. The obtained values of RSD and RE did not exceed 10.0% and 15.4%, respectively, with the variation not exceeding 15.4% for taxifolin 4′-*O*-sulfate and 15.0% for other tested compounds.

## 5. Conclusions

We have demonstrated for the first time the sulfation of the main silymarin constituents by human cytosolic sulfotransferases. We have found that (i) individual flavonolignans and taxifolin can be sulfated by multiple enzymes, (ii) most constituents can be converted to at least two relevant sulfation products, and (iii) all of these potential metabolites are monosulfates, some of which were identified as silybin A 20-*O*-sulfate, silybin B 20-*O*-sulfate, and isosilybin A 20-*O*-sulfate. With silymarin, the sulfation of flavonolignans and taxifolin was also demonstrated in human hepatocytes and the human intestinal cytosol. It is concluded that of the active enzymes, SULTs 1A1, 1A3, 1B1, and 1E1 might be involved in the hepatic or intestinal sulfation of silymarin constituents. We suggest that human sulfotransferases could be used to prepare authentic metabolites of flavonolignans and taxifolin, which can be helpful for further research on the pharmacology and metabolism of silymarin and its individual constituents.

## Figures and Tables

**Table 1:** Sulfation of flavonolignans and taxifolin by human tissue cytosols and human sulfotransferases.

Compound	*m/z* ^1^	t_R_ (min)	Semiquantitative Percentage ^2^										
			HLC	HIC	1A1*1	1A1*2	1A2	1A3	1B1	1C2	1C4	1E1	2A1
Silybin A (parent)	481.115	7.02	99.9	99.6	84.4	80.8	75.3	93.8	77.8	99.9	23.9	14.0	99.8
Sulfate (1)	561.069	5.67	0.0	0.1	3.7	4.3	1.3	4.3	0.6	0.1	5.0	85.4	0.1
20-*O*-Sulfate (2)	561.069	5.92	0.1	0.2	11.9	14.9	23.4	1.9	21.6	0.0	71.1	0.6	0.0
Sulfate (3)	561.069	6.08	0.0	0.1	0.0	0.0	0.0	0.0	0.0	0.0	0.0	0.0	0.1
Silybin B (parent)	481.115	7.19	99.9	99.1	96.3	97.1	90.3	54.9	66.1	99.9	58.0	13.7	99.9
Sulfate (1)	561.069	5.84	0.0	0.5	0.2	0.1	0.1	43.2	0.0	0.1	38.6	0.5	0.0
20-*O*-Sulfate (2)	561.069	6.11	0.1	0.3	3.5	2.8	9.4	1.8	33.8	0.0	3.2	85.8	0.1
Sulfate (3)	561.069	6.43	0.0	0.1	0.0	0.0	0.2	0.1	0.1	0.0	0.2	0.0	0.0
Isosilybin A (parent)	481.115	7.56	99.7	95.1	79.1	78.9	80.3	26.8	42.1	99.7	19.4	68.4	99.9
Sulfate (1)	561.069	6.23	0.1	1.4	16.3	16.6	6.0	17.1	0.0	0.2	0.2	0.1	0.0
20-*O*-Sulfate (2)	561.069	6.42	0.2	3.5	4.6	4.5	13.7	56.1	57.9	0.1	80.4	31.5	0.1
Isosilybin B (parent)	561.069	7.64	99.7	95.9	75.7	77.9	60.3	33.7	92.0	99.9	49.6	34.3	99.9
Sulfate (1)	561.069	6.33	0.1	1.5	13.4	12.9	36.9	31.2	3.7	0.1	37.6	9.2	0.0
Sulfate (2)	561.069	6.42	0.1	0.7	0.0	0.0	0.0	0.0	0.7	0.0	0.0	0.0	0.0
Sulfate (3)	561.069	6.57	0.1	1.9	10.9	9.2	2.8	35.1	3.6	0.0	12.8	56.5	0.1
Silychristin A/B (parent)	481.115	5.98 ^3^, 6.25 ^3^	99.9	95.7	81.9	83.6	96.0	12.4	49.6	99.6	13.4	10.0	100.0
Sulfate (1)	561.069	4.67	0.0	0.0	2.9	3.4	0.2	0.0	0.0	0.0	0.0	0.3	0.0
Sulfate (2)	561.069	4.97	0.0	0.0	0.0	0.0	0.9	0.0	0.0	0.4	0.0	0.0	0.0
Sulfate (3)	561.069	5.07	0.1	4.3	15.2	13.0	2.9	87.6	50.4	0.0	86.6	83.6	0.0
Sulfate (4)	561.069	5.25	0.0	0.0	0.0	0.0	0.0	0.0	0.0	0.0	0.0	6.1	0.0
Silydianin (parent)	481.115	6.19	99.2	98.9	8.3	8.7	78.2	95.1	99.9	99.8	99.7	9.3	100.0
Sulfate (1)	561.069	5.10	0.8	1.1	91.7	91.3	21.8	4.9	0.1	0.2	0.3	90.7	0.0
Taxifolin (parent)	303.050	4.41, 4.91 ^4^	99.7	85.7	83.8	87.5	41.2	0.4	33.5	99.9	0.6	59.5	100.0
Sulfate (1)	383.006	1.92	0.0	0.0	0.0	0.0	0.0	0.0	0.0	0.0	0.0	15.1	0.0
Sulfate (2)	383.006	2.97	0.0	0.0	0.0	0.0	5.7	0.3	0.0	0.1	0.7	16.1	0.0
4′-O-Sulfate (3)	383.006	3.69	0.0	0.1	0.0	0.0	0.8	0.2	0.0	0.0	0.1	1.0	0.0
Sulfate (4)	383.006	3.97	0.0	0.0	0.0	0.0	0.3	0.0	0.3	0.0	0.0	1.8	0.0
Sulfate (5)	383.006	4.21	0.3	14.2	16.2	12.5	52.0	99.1	66.2	0.0	98.6	4.3	0.0
Sulfate (6)	383.006	4.48	0.0	0.0	0.0	0.0	0.0	0.0	0.0	0.0	0.0	2.2	0.0

The tested flavonolignans and taxifolin (50 µM) were individually incubated for 30 min with pooled human liver cytosol (HLC), pooled human intestinal cytosol (HIC), and recombinant human SULTs 1A1*1, 1A1*2, 1A2, 1A3, 1B1, 1C2, 1C4, 1E1, and 2A1, and then analyzed by the UHPLC/MS method. Note: ^1^ *m/z* values for [M − H]^−^. ^2^ The semi-quantitative percentage values were evaluated on the basis of the peak area values. The sum of a given parent compound plus all of its sulfation products gives 100%. The values are means from three experiments. ^3^ The lower and higher t_R_ values were attributed to silychristin A and silychristin B, respectively [19]. Note: ^4^ t_R_ value attributed to a dihydroquercetin isomer with a configuration other than (2*R*,3*R*).

**Table 2:** Relative activity (%) of human sulfotransferases towards silymarin constituents.

Compound	1A1*1	1A1*2	1A2	1A3	1B1	1C2	1C4	1E1	2A1
Silybin A	18	21	28	7	25	0.1	76	100	0.2
Silybin B	5	3	11	62	44	0.2	53	100	0.1
Isosilybin A	24	23	19	81	69	0.3	100	35	0.1
Isosilybin B	38	34	62	100	12	0.2	69	85	0.2
Silychristin A/B	14	12	3	100	47	0.3	96	89	–
Silydianin	98	96	18	4	0.1	0.1	0.2	100	–
Taxifolin	7	5	30	100	38	0.1	82	14	–
Silymarin	10	11	44	58	60	0.2	55	100	0.1

The tested flavonolignans, taxifolin, and silymarin (50 µM) were individually incubated for 30 min with recombinant human SULTs 1A1*1, 1A1*2, 1A2, 1A3, 1B1, 1C2, 1C4, 1E1, and 2A1. The formation of flavonolignan monosulfates (*m/z* 561.069) and taxifolin monosulfates (*m/z* 383.006) was then examined using the UHPLC-MS method. The semiquantitative data show the relative activity (%) of individual sulfotransferases for the sulfation of the tested compounds, as evaluated on the basis of the peak area values. The enzyme producing the highest total amount of sulfates with a given compound representing 100% activity. The values are means from three experiments. The minus sign (–) indicates no sulfation activity.

**Table 3:** Chromatographic peaks found after sulfation of silymarin mixture by human hepatocytes, human tissue cytosols, and human sulfotransferases.

Peak No. ^1^	t_R_ (min)	*m/z*	Potential Metabolites ^2^
1	1.92	383.006	Taxifolin sulfate (1)
2	4.21	383.006	Taxifolin sulfate (5)
3	5.10	561.069	Silychristin sulfate (3), silydianin sulfate (1)
4	5.84	561.069	Silybin A sulfate (1), silybin B sulfate (1)
5	5.92	561.069	Silybin A 20-*O*-sulfate (2)
6	6.11	561.069	Silybin B 20-*O*-sulfate (2)
7	6.33	561.069	Isosilybin A sulfate (1), isosilybin B sulfate (1)
8	6.42	561.069	Isosilybin A 20-*O*-sulfate (2), isosilybin B sulfate (2)
9	6.57	561.069	Isosilybin B sulfate (3)

^1^ Peak numbers correspond to those in Figure 2, Figure 3 and Figure 4. ^2^ Numbers in brackets refer to metabolite numbers in Table 1.
